# Niche-Neutral Continuum Seems to Explain the Global Niche Differentiation and Local Drift of the Human Digestive Tract Microbiome

**DOI:** 10.3389/fmicb.2022.912240

**Published:** 2022-07-22

**Authors:** Hongju (Daisy) Chen, Zhanshan (Sam) Ma

**Affiliations:** ^1^Computational Biology and Medical Ecology Lab, State Key Laboratory of Genetic Resources and Evolution, Kunming Institute of Zoology, Chinese Academy of Sciences, Kunming, China; ^2^Center for Excellence in Animal Evolution and Genetics, Chinese Academy of Sciences, Kunming, China; ^3^College of Mathematics, Honghe University, Yunnan, China

**Keywords:** unified neutral theory of biodiversity (UNTB), niche-neutral hybrid model (NNH), multi-site neutral model (MSN), digestive tract (DT) niche differentiation, species-level neutrality analysis

## Abstract

The human digestive tract (DT) is differentiated into diverse niches and harbors the greatest microbiome diversity of our bodies. Segata et al. (2012) found that the microbiome of diverse habitats along the DT may be classified as four categories or niches with different microbial compositions and metabolic potentials. Nonetheless, few studies have offered theoretical interpretations of the observed patterns, not to mention quantitative mechanistic parameters. Such parameters should capture the essence of the fundamental processes that shape the microbiome distribution, beyond simple ecological metrics such as diversity or composition descriptors, which only capture the manifestations of the mechanisms. Here, we aim to get educated guesses for such parameters by adopting an integrated approach with multisite neutral (MSN) and niche-neutral hybrid (NNH) modeling, *via* reanalyzing Segata’s 16s-rRNA samples covering 10 DT-sites from over 200 healthy individuals. We evaluate the relative importance of the four essential processes (drift, dispersal, speciation, and selection) in shaping the microbiome distribution and dynamics along DT, which are assumed to form a niche-neutral continuum. Furthermore, the continuum seems to be hierarchical: the selection or niche differentiations seem to play a predominant role (> 90% based on NNH) at the global (the DT metacommunity) level, but the neutral drifts seem to be prevalent (> 90% based on MSN/NNH) at the local sites except for the gut site. An additional finding is that the DT appears to have a fifth niche for the DT microbiome, namely, Keratinized gingival (KG), while in Segata’s original study, only four niches were identified. Specifically, in Segata’s study, KG was classified into the same niche type including buccal mucosa (BM), hard palate (HP), and KG. However, it should be emphasized that the proposal of the fifth niche of KG requires additional verification in the future studies.

## Introduction

The digestive tract (DT) of humans is differentiated into diverse habitats or niches and harbors the greatest microbiome diversity of our bodies. For example, Segata et al. (2012) found that the microbial communities of diverse habitats along the DT can be recognized as four distinctive types, each is different not only in community composition, but also in metabolic potentials or functionalities (niches). However, existing studies were focused on effective characterizations of the community structure and functions, and few have offered theoretical interpretations of the observed patterns, especially with quantitative mechanistic parameters. Such mechanistic parameters should capture the essence of the fundamental processes that shape the microbiome distribution and dynamics, beyond simple ecological metrics such as diversity or composition descriptors, which only capture the manifestations (outcome) of the processes, rather than the processes (mechanisms) *per se*. We aim to estimate such parameters by adopting an integrated approach with the neutral theory of biodiversity and niche-neutral hybrid (NNH) modeling by reanalyzing Segata et al.’s (2012) 16s-rRNA datasets of 10 DT sites from over 200 healthy individuals.

We subscribe to the recent syntheses of community ecology and biogeography by Vellend (2010) in macrobial and Hanson et al. (2012) in microbial ecology. According to their syntheses, the four main processes (drift, dispersal, speciation, and selection) constitute the major underlying mechanisms that drive community diversity patterns and drive biogeography. The syntheses in community ecology (Vellend, 2010) and microbial biogeography (Hanson et al., 2012) were inspired by a similar synthesis of the four essential processes (drift, mutation, gene flow, and selection) in population genetics, which forms the modern theoretical foundation of population genetics. In the ecological synthesis, it is the speciation and dispersal that adds species to communities, and then the drift, selection, and ongoing dispersal mold the community composition and diversity. The primary objective of this study was to investigate the relative importance of the four processes in shaping community diversity patterns of the human DT microbiome, and a by-product from achieving the objective is a mechanistic interpretation for the global function (metabolic potentials) or niche differentiation along the DT, as observed by Segata et al. (2012). Furthermore, we demonstrate two applications of the niche-neutral modeling in detecting possible niche differentiations and in estimating the microbial transfer (dispersal) within the DT. This study is to our knowledge the first comprehensive examination of the four main ecological processes in shaping diversity patterns along the DT.

We recognize the debates surrounding Hubbell’s unified neutral theory of biodiversity and biogeography (UNTB) (e.g., Hubbell, 2001; Purves and Pacala, 2005; Alonso et al., 2006; McGill et al., 2006; Kadmon and Allouche, 2007; Chisholm and Pacala, 2010; Rosindell et al., 2011; Tang and Zhou, 2013; Avershina et al., 2014; Matthews and Whittaker, 2014; Hammal et al., 2015; Burns et al., 2016; Li and Ma, 2016, 2019; Dai et al., 2017; Ma, 2021a,b) during the past decade. While the UNTB maintains that stochastic drifts play driving roles in community assembly and diversity maintenance, natural communities are also structured by stabilizing niche differences and competitive asymmetries among species, which typically generate distinctly non-neutral communities (Gilbert and Levine, 2017). Consequently, the level of neutrality exhibited by a community can act as a measure of *drift effect* (stochasticity) in the community. In fact, the UNTB also takes into account the speciation and dispersal (migration) (Hubbell, 2001). Together, neutral theory can act as a null model, to the minimum, to offer an educated guess for the three of the four processes (excluding selection) in Vellend’s (2010) and Hanson et al.’s (2012) syntheses.

To quantify the effect of *selection*, which is the difference in the deterministic fitness between individuals from different species, and can also be treated as the deterministic interactions among species and between species and their environments (Vellend, 2010), we apply NNH models. There are several NNH models in the literature (e.g., Tilman, 2004; Ofiteru et al., 2010; Stokes and Archer, 2010; Jeraldo et al., 2012; Pigolotti and Cencini, 2013; Tang and Zhou, 2013; Fisher and Mehta, 2014; Kalyuzhny et al., 2014; Matthews and Whittaker, 2014; Noble and Fagan, 2015), which can be equally or even more powerful than the neutral theory. We chose Tang and Zhou’s (2013) NNH model for the following three reasons: (*i*) The NNH model is well suited to the hierarchical metacommunity of the DT where 10 sites (i.e., local communities) are sampled. (*ii*) One of the hotly debated topics of neutral theory is whether niche and neutral processes can generate similar patterns but *via* different mechanisms or interact to jointly generate the observed diversity patterns, or the patterns predicted by the neutral model are robust to niche structure (e.g., Chisholm and Pacala, 2010; Tang and Zhou, 2013; Hammal et al., 2015). Tang and Zhou’s (2013) NNH model can help to explore this mechanistic question. (*iii*) The NNH model structure is not only consistent with the data structure of the DT microbiome, but also consistent with the model structure of the multi-site neutral (MSN) model, which was first developed by Harris et al. (2017) as explained below.

Harris et al. (2017) developed a computationally highly efficient hierarchical Dirichlet process (HDP)-based approximation of Hubbell’s (2001) neutral theory. The approach was implemented with Bayesian machine learning algorithm, which makes it possible to efficiently and simultaneously estimate the migration among large number of sites (i.e., truly multisite metacommunity setting). Since the problem of estimating migration rates in multi-site setting is computationally intractable, Harris et al.’s (2017) multi-site neutral (MSN) model is a major advance in the field and we adopt their models to test the neutrality in this study. As mentioned previously, the data structure of the DT microbiome samples is consistent with the model structures of both the MSN and NNH. This consistency is critical for the integrated analysis of the relative importance of the four processes in shaping the diversity patterns along the human DT. The main objective of this study is therefore to apply the MSN (Harris et al., 2017) and NNH (Tang and Zhou, 2013) models as the duo approaches for characterizing the niche-neutral continuum, which is assumed to be primarily shaped by the balance between stochastic neutral drifts and deterministic selection.

## Methods

### Multi-Site Microbiome Datasets of the Digestive Tract

Segata et al. (2012) showed that the DT microbiome formed four distinctive types based on similar community compositions: (1) buccal mucosa (BM), keratinized gingiva, hard palate (HP); (2) saliva, tongue, tonsils, throat; (3) sub-gingival plaques and supra-gingival plaques; and (4) stool. In short, the authors took a total of 2,105 samples from 10 sites of over 200 individual subjects, which were sequenced by 16s-rRNA amplicon sequencing, and generated operational taxonomic unit (OUT) tables of the DT microbiome, which we use in this study to fit the MSN and NNH models. Further information on the DT microbiome dataset is referred to Segata et al.’s (2012) original research. It should be noted that, while they associate the 10 sites to the DT microbiome, it is only the stool samples that capture the gut microbiome. The other sites are in the mouth and throat.

In the focal study, we treat the 10 DT sites of each individual subject as 10 local communities, which constitute a metacommunity as a whole. In other words, the DT of each individual subject hosts a metacommunity consisting of 10 local microbial communities. Since the original datasets reported by Segata et al. (2012) did not identify the samples of DT microbiomes to individual subjects (possibly to protect privacy), and therefore, we could not simply fit the MSN or NNH model directly on individual subject basis. The downloaded data from Segata et al. (2012) consist of a vector of approximately 200 samples (from 200+ individuals) for each DT site and 10 vectors in total for the whole cohort of 200+ individuals. To obtain the reliable results of fitting the MSN/NNH models, we randomly selected 10 samples from the cohort vectors, one sample from each of the 10 vectors, to fit a MSN/NNH model. The random selection was repeated for 1,000 times, that is, we built 1,000 meta-community models to test the neutrality at both local- and meta-community levels. With the 1,000 times of re-sampling, 1,000 sets of MSN/NNH models were constructed and tested. We used the percentage of passing the MSN (neutrality) or NNH (niche differentiations) from the 1,000 sets of MSN/NNH models to judge the neutrality/niche differentiations. For the mathematical and computational details of fitting the MSN and NNH models, readers are referred to Tang and Zhou (2013) and Harris et al. (2017). A brief introduction on both the models is presented in the Online Supplementary Information (OSI).

### Hubbell’s UNTB and Vellend–Hanson Synthesis for Community Ecology

Hubbell’s (2001) UNTB conceptually distinguishes the local community dynamics from metacommunity dynamics, but both are driven by similar neutral process. Metacommunity dynamics are controlled by two quantities: speciation probability and reproduction rate of an individual. Diversity of the local communities is maintained by immigration from the metacommunity, but no speciation is assumed to occur unlike in the metacommunity. With these assumptions, Hubbell’s neutral theory was formulated as a master equation (a stochastic differential equation), with a solution that is a probability distribution (sampling formula), which can be compared against species abundance distribution (SAD) obtainable by sampling ecological communities.

While Hubbell’s neutral theory of biodiversity was inspired by Kimura’s neutral theory of molecular evolution in population genetics, Vellend’s (2010) and Hanson et al.’s (2012) syntheses of community ecology were also inspired by similar synthesis in population genetics based on genetic drift, mutation, dispersal, and selection. According to Vellend (2010) and Hanson et al. (2012), species are added to communities *via* speciation (which generates new species) and dispersal (the movement of organisms across space). The relative abundances of these species are then shaped by *drift* and *selection*, as well as ongoing dispersal. Consequently, these four processes constitute the underlying forces that drive microbial community dynamics (Vellend, 2010) and biogeographic patterns on inseparable ecological and evolutionary scales (Hanson et al., 2012). Hubbell’s neutral theory of biodiversity takes into accounts the effects of *drift*, *dispersal* and *speciation*, but the theory ignores selection, the only missing aspect in the four processes synthesis (Rosindell et al., 2011). Since it is formulated as a testable null model, the level of non-neutrality may be considered as a proxy for selection, at least partially. When selection is relatively strong and the community size is large, selection will cancel any effects of drift. However, when selection is relatively weak and the community size is small, drift can overrule the effects of selection. Between these two extremes, selection drives some community outcomes more likely than others, but it does not guarantee any particular outcome (Nowak, 2006; Vellend, 2010). In other words, community states constitute a continuum driven by deterministic selection and stochastic drift forces, and the selection–drift interactions may reach a dynamic equilibrium (balance) that determines where a particular community state resides on the continuum at a specific time-space-environment setting. We term this integration of neutral and niche effects (theories) as niche-neutral continuum.

### The Niche-Neutral Hybrid Model

Given the observation that the human DT is differentiated into diverse niches such as those represented by the different metabolic potentials or functionalities, we postulate that a pure neutral-theoretic model is unlikely to describe the DT metacommunity satisfactorily. Instead, a NNH model is more likely to succeed. Tang and Zhou’s (2013) NNH model was designed to investigate the relative importance of niche differentiation and neutral processes that may shape the SAD (species abundance distribution) in spatially discrete communities. They assumed that a semi-isolated community consists of *K* non-overlapping niches. Within each niche, a number of species follow their own neutral rules independent of the other *K*-1 niches. For example, here, we assume that the different sites along the DT can be represented as different niches, differing in their suitability for different microbes. Within a site (niche), microbes follow their neutral rules.

Tang and Zhou’s (2013) NNH model was derived by incorporating the niche differentiations into Volkov et al.’s (2007) multi-site neutral model. Specifically, the per capita birth to death rates (*x* = *b*/*d*) and immigration parameter (γ) vary among species from different niches. In the case of our multi-site DT microbiome datasets, we treat each site as a niche occupied by a local microbial community. A Chi-square test is performed by comparing the predicted and observed numbers of species for each corresponding abundance level. The *p*-value of Chi-square test is then utilized to determine whether or not the NNH model is a good fit for a set of microbial communities sampled from the multiple microbiome sites of the DT of an individual. Specifically, at the metacommunity level, if *p*-value > 0.05, then the metacommunity of DT microbiome satisfies the NNH and the metacommunity dynamics should be driven by both niche (at the whole metacommunity scale) and neutral processes (at local community scale), which also implies that the metacommunity as a whole does not satisfy the neutral theory, but within each niche (DT site), the local community is neutral. If *p*-value < 0.05, the metacommunity does not satisfy the NNH, which means that within each site (niche), the local community is non-neutral either, and the metacommunity assembly may be influenced by the niche process alone.

### The Multi-Site Neutral Model

While niche differentiations are certainly important in shaping the DT microbiome diversity patterns, neutral processes can still be important, given the localization of various metabolic potentials that may create relatively homogenous local host environment for site-specific microbiomes. That is, each site may have site-specific habitat to host special functional groups of microbes. We therefore resort to the neutral theory model to assess and interpret the observed patterns of DT microbiome. Specifically, Harris et al.’s (2017) MSN model, which improves the fitting of Hubbell’s (2001) classic neutral model, is advantageous for our objectives.

A fully general case of fitting multiple sites UNTB with variable immigration rates among sites is computationally intractable even for the moderate number of sites, and approximate algorithms must be utilized (Harris et al., 2017). Harris et al. (2017) developed an efficient Bayesian framework by approximating the neutral models with the HDP. Harris et al. (2017) approximation captures the essential elements of the UNTB, i.e., neutrality, finite populations, and multiple panmictic geographically isolated populations linked by relatively rare migration. With Harris et al.’s (2017) HDP-MSN model, it is possible to distinguish between neutral local community (given a non-neutral metacommunity) and the full UNTB (where the metacommunity also assembles neutrally). Hence, the neutrality is tested at the metacommunity and local community level. Besides the original publication of the MSN model (Harris et al., 2017), a simplified outline of the computational procedure to implement the MSN model, including the correction to a minor typo error that influences the interpretation of the model results, can also be found in Ma (2022).

## Results

### Community-Level Niche-Neutral Continuum Analysis With MSN and Niche-Neutral Hybrid Models

**FIGURE 1:**
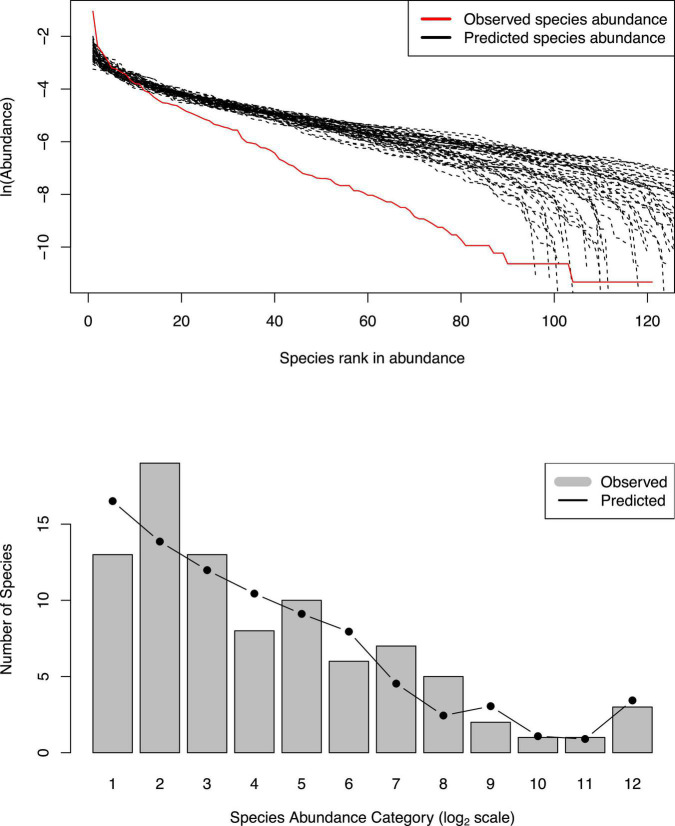
Examples showing the fittings of the MSN and NNH models to DT microbiomes: (*i*) the top graph from ^#^275 re-sampling showing a failed fitting to MSN model; (*ii*) the bottom graph from ^#^1 re-sampling showing a successful fitting to NNH model.

Supplementary Table 1 in the OSI exhibited the full (1,000 sets) test results of the MSN model, and Table 1 below exhibited the 6 examples excerpted from Supplementary Table 1. The parameters involved in the MSN neutral testing were explained as a part of table legend at the end of Table 1. Figure 1 (top graph) shows an example of fitting the MSN model with the DT microbiome datasets.

**TABLE 1:** The selected test results of fitting Harris et al.’s (2017) HDP-MSN (hierarchical *Dirichlet* process approximated multi-site neutral model) to the microbiomes of human digestive tract (DT), excerpted from Supplementary Table 1 in the OSI where the full fitting results from 1000 times of re-sampling were exhibited *,**.

*ID*	*L*_*O*_	θ	*M*-value	Metacommunity (Body)				Local Community (DT site)			
					
				*L*_*M*_	*N*_*M*_	*N*	*P*_*M*_	*L*_*L*_	*N*_*L*_	*N*	*P*_*L*_
1	–2413.840	30.442	21.788	–2990.259	2466	2500	0.986	–2592.323	2405	2500	0.962
2	–2637.699	35.494	17.573	–3090.535	2402	2500	0.961	–2823.226	2379	2500	0.952
3	–2953.500	33.751	27.785	–3469.276	2393	2500	0.957	–3152.171	2422	2500	0.969
10	–2863.917	43.612	17.689	–3423.708	2460	2500	0.984	–3085.267	2433	2500	0.973
27	–2472.904	38.563	13.240	–2889.208	2426	2500	0.970	–2655.564	2402	2500	0.961
67	–3549.417	41.429	24.700	–4315.819	2469	2500	0.988	–3807.775	2432	2500	0.973
…	…	…	…	…	…	…	…	…	…	…	…
**Mean**	–2748.406	35.754	22.990	–3321.013	2433	2500	0.973	–2917.173	2329	2500	0.932

*N = 2,500 is the number of Gibb samples selected from 25,000 simulated communities (i.e., every tenth iteration of the last 25,000 Gibbs samples, while the first 25,000 simulations were discarded as the burn-in), it is chosen to compute the pseudo-p-value below for conducting the neutrality test. L_0_ is the actual log-likelihood, which is compared with the log-likelihood of each simulated community. θ is the median of biodiversity parameters computed from 25,000 times of simulations. M-value is the average medians of the migration rates of local communities in each metacommunity, also computed from 25,000 times of simulations. L_M_ is the median of the log-likelihoods of the simulated neutral metacommunity samples; N_M_ is the number of simulated neutral metacommunity samples with their likelihoods satisfying L ≤ L_0_ (where L is the simulated, and L_0_ is the actual likelihood). P_M_ = N_M_/N is the pseudo-P-value for testing the neutrality at metacommunity level: if P_M_ > 0.05, the metacommunity is indistinguishable from what is predicted by the MSN model. L_L_ is the median of the log-likelihoods of the simulated local community samples, and N_L_ is the number of simulated local community samples with their likelihoods not exceeding the L_0_. P_L_ = N_L_/N, is the pseudo-p-value for testing the neutrality at the local community level: if P_L_ > 0.05, the local community is indistinguishable from what is predicted by the MSN model. **Both P_M_ and P_L_ have been corrected from the typo/errors in Harris et al.’s (2017) computational outputs when summarized from Supplementary Table 1.

Supplementary Table 2 in the OSI exhibited the full (1,000) test results of the NNH model, and Table 2 below exhibited the 6 examples excerpted from Supplementary Table 2. The parameters involved in the NNH testing are explained as a part of table legend at the end of Table 2. Figure 1 (bottom graph) also shows an example of fitting the NNH model with the DT microbiome datasets.

**TABLE 2:** The selected test results of fitting Tang and Zhou’s (2013) NNH (niche-neutral hybrid) model to the microbiomes of human digestive tract (DT), excerpted from Supplementary Table 2 in the OSI where the full fitting results from 1,000 times of re-sampling were exhibited*.

ID	*J*	*S*	θ	*m*	*x*	γ	*R* ^2^	χ^2^	*P*-value	*N*	*N^pass^*	%*^(pass)^*
1	4086.333	29.333	6.200	0.000	0.849	1.235	0.812	8.336	0.683	3	3	100
2	8916.000	40.667	6.531	0.000	0.794	1.422	0.904	12.464	0.490	6	5	83.3
3	7209.000	49.500	9.425	0.000	0.825	1.292	0.879	15.320	0.224	6	6	100
10	9304.800	48.200	15.775	0.000	0.767	0.957	0.877	21.909	0.039	5	4	80
27	10411.000	41.286	9.691	0.000	0.761	1.426	0.964	27.185	0.012	7	6	85.7
67	19083.625	53.500	12.438	0.000	0.797	1.211	0.946	194.652	0.000	8	7	87.5
…	…	…	…	…	…	…	…	…	…	…	…	…
**Mean**	10306.377	47.428	9.366	0.000	0.796	1.377	0.915	24.850	0.547	5.921	5.676	95.862

*J: The average number of individuals per niche (local community) in each metacommunity, S: the average species numbers per niche (local community) in each metacommunity, θ: the average fundamental biodiversity parameter per niche (local community) in each metacommunity, m: the average of the migration coefficients, x: the average of the birth to death ratio, **γ**: the average of the migration rate, R^2^: the goodness-of-fitting index, χ^2^-value: the χ^2^-value of chi-squared test for observed value against predicted value, p-value for the χ^2^-test; when p-value > 0.05, the metacommunity is indistinguishable from what is predicted by the NNH model. The last two columns are the number and percentage of local communities (niches) that passed the local neutrality test.

**FIGURE 2:**
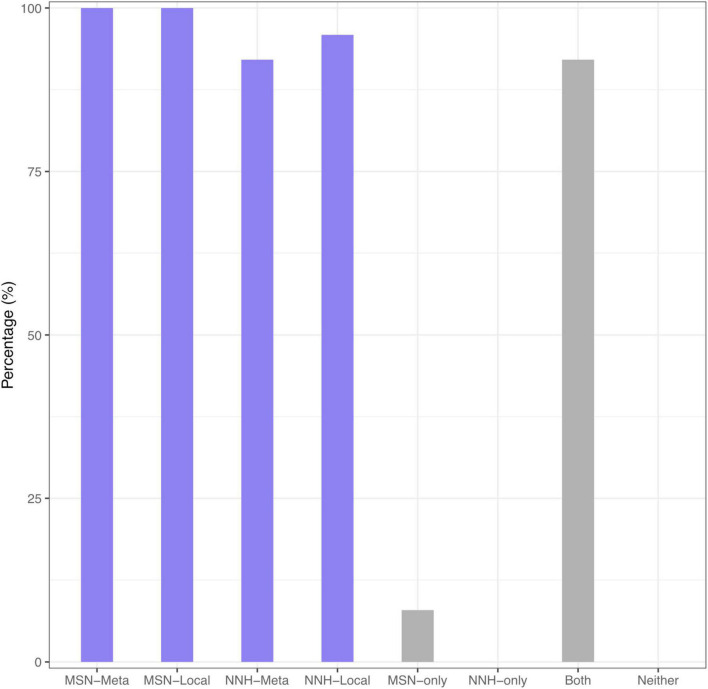
The passing percentage from testing the MSN/NNH models: (*i*) The left block (purple) shows the percentage for passing MSN at metacommunity, MSN at local community, NNH at metacommunity, and NNH at local community level, respectively; (*ii*) The right block (gray) shows the percentage for passing MSN-only, NNH-only, both MSN and NNH, and neither MSN nor NNH, all fitted at the metacommunity level. The two blocks were displayed with different objectives: the left was from the perspective of comparing the performance of MSN vs. NNH, and the right was from evaluating the overlap or specificity of the MSN vs. NNH models.

Supplementary Table 3 (also displayed in the left side of Figure 2) summarized the passing rates of MSN and NNH at both local and metacommunity levels, respectively, summarized from Supplementary Tables 1, 2. Regarding the MSN model, the passing rate of neutrality test is 100% both at metacommunity and at local community levels. Regarding the NNH model, at the metacommunity level, 92.1% meta-communities are niche-differentiated, and at the local-community level, 95.9% local communities are neutral. That is, both MSN and NNH models support the inference that, at the local community (individual DT site) level, the patterns are indistinguishable from neutral assumptions. In other words, neutral processes are predominant at the local community level. However, at the metacommunity level, while the MSN model still supports the neutral assumptions, the NNH model suggests niche differentiations. Given the contradictory inferences from the MSN and NNH, we tend to accept the finding from the NNH model since the MSN is formulated as a null model based on more idealized or less realistic assumptions (Hubbell, 2001; McGill et al., 2006; Chisholm and Pacala, 2010; Rosindell et al., 2011; Hammal et al., 2015; Ma, 2020, 2021a,b). According to the NNH inferences, the DT, i.e., the total habitat for the metacommunity of an individual, is differentiated into multiple niches (sites or local communities), within which neutral drifts play a predominant role. This is also supported by Segata et al.’s (2012) observation that DT sites can be classified into different types, not only different in community composition but also in metabolic functions. Further justifications of our siding with the NNH results (i.e., against the MSN) at the metacommunity level are extended in the section “Discussion.”

**FIGURE 3:**
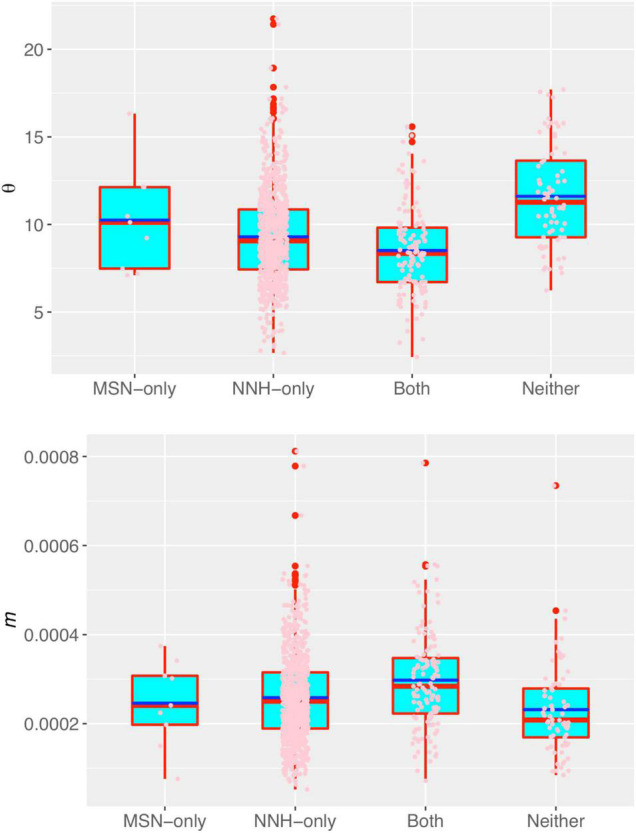
The box plots of fundamental biodiversity number (θ) (the top) and the fundamental dispersal number (migration probability: *m*) of the human DT microbiome, both estimated with the NNH model. Three standard summary statistics of the parameters (θ) and (*m*), including the first quartile (lower edge of the rectangle), median (the inside segment), third quartile (upper edge of the rectangle) were displayed, respectively. The “whiskers” above and below the box (rectangle) show the location of the minimum and maximum. The inter-quartile range (IQR) (showing the range of variation) is displayed by the height of the box; and the median shows the typical value. Outliers (<3×IQR or >3×IQR) are displayed outside the box.

In Supplementary Table 4, we tested the statistical distributions of the key parameters of the MSN and NNH models, and it was found that all key parameters satisfactorily fitted to the power law statistical distribution, but failed to fit the normal (Gaussian) distribution. The fitting failure of normal distribution and success of power law distribution indicated that individuals are rather heterogeneous in their DT microbiomes. The power law distribution is highly skewed with a long tail, indicating extreme heterogeneity among individuals in their key parameters. That is, the DT microbiome of most individuals has small parameter (e.g., migration probability) values, and a small number of individuals have disproportionally large parameter values. In addition, there is not an average “Joe” who can represent majority of the individuals according to the so-termed no-average property of power law. In other words, intra-body distribution of DT microbiome is highly personalized, which is a well-known observation in microbiome research (HMP Consortium, 2012), but our analysis presented a mechanistic interpretation to the observed phenomenon. The high heterogeneities were also exhibited by the fundamental biodiversity number (θ) and fundamental dispersal number (migration probability *m*) (Figure 3).

Supplementary Table 5 (*also* displayed in the right side of Figure 2) shows the percentages of passing tests with MSN and NNH, exclusively and inclusively, i.e., passing MSN only, NNH only, both MSN and NNH, neither MSN nor NNH. This table was intended to address the possible overlap between MSN and NNH models. It turned out that there were 92% of cases that both MSN and NNH models were fitted successfully, and consequently, the other categories were negligible. Given the previously stated argument that the MSN model was based on more idealized assumptions, we follow the findings from the NNH model.

Figure 3 displays the fundamental biodiversity number (θ) and fundamental dispersal number (*m*: migration probability) for each of the four category listed in Supplementary Table 5. Note that both θ and *m* displayed in Figure 3 were estimated with the NNH model, given that NNH successfully fitted to more than 90% of the metacommunities and 95% local communities (Supplementary Table 3 and Figure 2). A remarkable property of both the parameters is their high heterogeneities among individuals (or among different metacommunities). That is, the DT microbiomes of individuals are highly personalized.

### Quantification of Niche Differentiations and Detection of a Fifth Niche

In previous subsections, we demonstrated that the abundance and distribution of human DT microbiomes could be described with the niche-neutral continuum. Our analysis revealed that the continuum is hierarchical and that selection or niche differentiation plays a predominant role (> 90%) at the metacommunity level, but the neutral forces play a predominant role (> 90%) at local site (of DT) level (Figure 2 and Supplementary Tables 3, 5). These quantitative derivations confirmed the generally qualitative findings from Segata et al.’s (2012) original study—the microbiota of diverse habitats along the DT can be distinguished as four types; each is different not only in community composition, but also in metabolic potentials (functionalities or niches). Nevertheless, as demonstrated below, our quantitative results can actually be harnessed to correct an inaccuracy of the four niches identified in Segata et al.’s (2012) original study. That is, there may be *five*, rather than *four*, niches in the human DT microbiomes.

**FIGURE 4:**
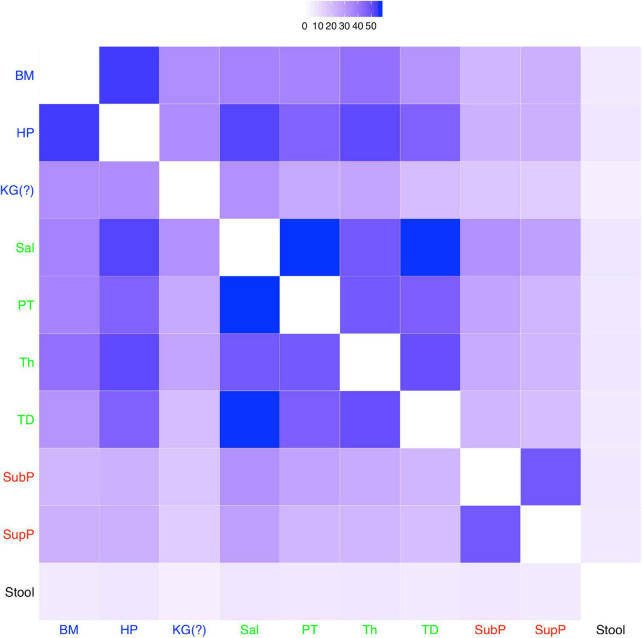
A heatmap showing the *M*-value (the average medians of the *migration rates* of local communities in each metacommunity) of the 10 sites: the larger the *M*-value is (the deeper the color in the heatmap is), the higher the similarity between the DT sites (see Supplementary Table 3 for detailed results of other MSN parameter). A fifth niche, i.e., KG (keratinized gingival), together with the four niches (the four types of DT microbiota, i.e., “BM-HP-KG,” “Sal-PT-Th-TD,” “SubP-SupP,” and “Stool,” colored differently, and originally discovered by Segata et al. (2012)) emerged as five clusters here. The fifth niche (KG) was originally classified into the “BM-HP-KG” type by Segata et al. (2012).

To illustrate the process for identifying the possible niches in the DT microbiomes, we fitted the MSN model to the pair-wised (pair of sites) microbiome samples (from the 10 DT sites) across approximately 200 healthy individuals, and a total of 45 possible MSN models (corresponding to the 45 possible groups of pair-wised site samples) were obtained (refer to Supplementary Table 6). Figure 4 shows the heatmap drawn based on the MSN parameter *M*, which represents for the average median of migration rates within a metacommunity. Obviously, the larger the *M* is, the more similar the local communities (within the metacommunity) are. This is because the exchange of individuals through migration should lead to homogeneity (similarity) within the metacommunity. When applying this information for distinguishing among different niches, *within-niche* similarity should be higher and *between-niche* similarity should be lower.

As illustrated in Figure 4, the four niches can be easily identified: (*i*) stool; (*ii*) sub-gingival plaques and supra-gingival plaques (SubP and SupP); (*iii*) tongue dorsum (TD), throat (TH), palatine tonsils (PT) and saliva (Sal); and (*iv*) hard palate (HP) and buccal mucosa (BM). However, the site of *keratinized gingiva* (KG), which was originally classified into the same niche with HP and BM, may not be easily classified into any of the four niches. The similarity between KG and HP (or BM) is obviously significantly lower than the similarity between HP and BM (the other two sites of the original 3 sites niche in Segata et al., 2012 classification), as illustrated by the lighter color in the heatmap (Figure 4). In fact, the site KG is not similar to any of the other 9 sites, as illustrated by the light color in the heatmap across all other sites. Therefore, KG should be classified as a standalone niche, just like stool, rather than as belonging to the same niche with HP and BM. The justification for separating KG as a standalone (separate) niche is also exhibited obviously in Table 3: the *M*-value for KG is 21.485 vs. 29.209 (BM) or 34.184 (HP). In addition, although KG appears to have similar level of *M*-value with SubP and SupP, they have rather different fundamental biodiversity numbers (θ), again supporting a standalone niche for KG. In fact, the five niches classification is also supported by the values of θ of the 10 different DT sites (Table 3).

**TABLE 3:** The mean MSN (multi-site neutral) model parameters of 10 DT (digestive tract) sites: the fundamental biodiversity number (θ) and fundamental dispersal number (*M*).

Five niches	Site ID	MSN model parameter	
		
		θ	*M*
Buccal mucosa (BM) and Hard palate (HP)	BM	36.329	29.209
	HP	36.133	34.184
Keratinized gingival (KG)	KG	34.770	21.485
Saliva (Sal), Palatine tonsils (PT), Throat (TH), and Tongue dorsum (TD)	Sal	36.824	35.622
	PT	36.703	31.897
	Th	36.472	32.535
	TD	34.558	30.277
Sub-gingival plaques (SubP) and Supra-gingival plaque (SupP)	SubP	40.228	22.991
	SupP	40.203	21.451
Stool (Gut)	Stool	142.229	6.396

### Oral-Gut Microbiome Dispersal

**FIGURE 5:**
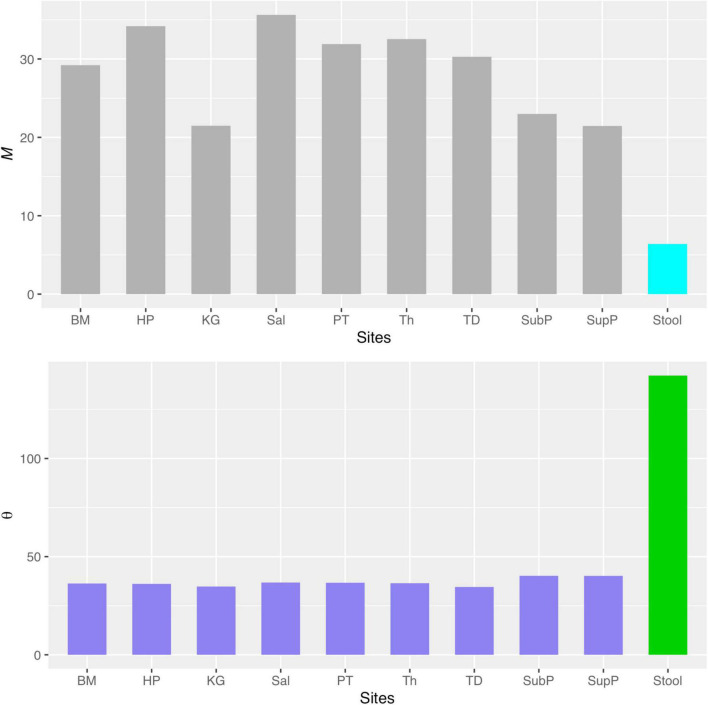
The comparisons of migration site (*M*) and fundamental biodiversity numbers (θ) between oral microbiomes (the left nine sites from BM to SupP) and gut (stool) microbiome.

Besides identifying potential niche as demonstrated previously, another potential application of the MSN model can be to estimate the transfer (dispersal or migration) between microbiome sites such as the dispersal between oral and gut microbiome. In the case of this study, we can use the MSN model parameter to estimate the transfer (dispersal) between oral sites and the gut (stool) microbiome. As shown in Figure 5 (the top graph), the migration rate (*M*-value) between any of the oral sites and gut (stool) site is significantly lower than the migration rates among oral sites. Similarly, Figure 5 (the bottom graph) also shows that the fundamental biodiversity number (θ) of gut (stool) is significantly higher than the θ of oral sites. The same information is also exhibited in Table 3, in which the biodiversity number (θ) of the gut is approximately 142, while the θ for each of the oral sites is smaller than 42, i.e., more than 3 times of differences. The average migration rate (*M*) from any oral site to the gut (stool) site is 6.4 approximately, while the average *M* between the oral sites exceeds 21, again more than 3 times of the differences. Therefore, the MSN model parameters can be harnessed not only to quantify the microbial dispersal from oral to gut, but also to compare the dispersal among different sites. That the dispersal from oral to gut is less than 1/3 of the dispersal between oral sites is a new finding, which is reasonable in our opinion. Such kind of information is difficult to obtain experimentally. It is also difficult to infer from other ecological models/metrics such as routinely computed diversity indexes or more sophisticated community compositional analysis (e.g., Ma and Ellison, 2019; Ma et al., 2019). The significantly higher biodiversity number (θ) with gut confirms a well-recognized consensus that the gut hosts the greatest microbiome diversity in our bodies.

## Discussion

By leveraging the truly multi-site DT microbiome datasets originally reported by Segata et al. (2012), which collected the 16s-rRNA samples covering 10 DT sites from over 200 healthy individuals, and by utilizing Harris et al.’s (2017) multi-site neutral (MSN) model and Tang and Zhou’s (2013) NNH model, we aim to evaluate the relative significance of the four processes, i.e., drift, dispersal, speciation, and selection in shaping the distribution patterns of the bacteria along the DT, besides testing the performance of those neutral-theoretic models. According to Vellend (2010) and Hanson et al. (2012), the four processes (drift, speciation, dispersal, and selection), similar to the four processes (drift, mutation, gene flow, and selection) that form the modern synthesis in population genetics, constitute the mechanisms underlying the community diversity patterns (the core of biogeography) and community dynamics. According to the synthesis, it is the speciation and dispersal that add species to communities, and the drift, selection, and ongoing dispersal that shape the community composition and diversity along the DT. The “product” of the four processes is a *niche-neutral continuum*. At different localities of the continuum, the relative importance of niche vs. neutral (or deterministic selection vs. stochastic drifts) can be different. Quantifying the above-stated Vellend–Hanson synthesis has been challenging given its highly qualitative nature, and here, we try to put our findings into the framework of the synthesis. We treated the microbiomes along the DT of an individual as a metacommunity consisting of 10 local communities (corresponding to 10 DT sites sampled). The first point we postulated and confirmed with the MSN/NNH duo analysis is that the niche-neutral continuum is hierarchical. Globally, the niche differentiations as suggested by the NNH analysis appear to be predominant (> 90%) and we postulated that the selection is in effect. This finding is consistent with Segata et al.’s (2012) original study that identified four specific microbiota types harbored at different DT sites. Locally, neutral processes seem to be predominant as suggested by both MSN and NNH models (NNH > 90%, MSN = 100%). These numbers cross-verified the hybrid effects of selection and neutral drifts along the DT as revealed by the MSN/NNH duo. In summary, this study generated two pieces of supporting evidence to Vellend–Hanson synthesis with the human DT microbiome as a model system. First, the hierarchical niche-neutral continuum demonstrated the coexisting selection, drift, and dispersal effects. Second, the numbers from MSN/NNH models could offer educated guesses for the relative importance of the four processes.

In the remainder of this article, we briefly discuss two issues often associated with the testing of neutral theory, which we could not circumvent and actually turned out becoming the limitations of this study. One is the lack of full specificity or non-unique fitting of neutral or NNH models in fitting SAD datasets—multiple models with potentially different ecological assumptions may fit to same dataset equally well, leading to uncertainty or even dilemma in mechanistic inferences. This issue has been known shortly after the neutral theory for biodiversity was proposed and is arguably one of the strongest criticisms of using SAD for mechanistic inference (e.g., Purves and Pacala, 2005; McGill et al., 2006; Kadmon and Allouche, 2007; Chisholm and Pacala, 2010; Tang and Zhou, 2013; Hammal et al., 2015). In the case of our study, the overlap between the MSN and NNH has reached an apparently unfortunate level of > 90%. However, a careful examination of the models and their test results reminds us that, at the local community level, the model assumptions and test results of both MSN and NNH are totally consistent—neutrality exceeding 90%. At the metacommunity level, the assumptions are different, the MSN assumes neutrality and the NNH assumes niche differentiations, and we sided with the NNH in consideration that the NNH assumption was more realistic and the MSN assumption of neutral species was more idealized. Of course, Segata et al.’s (2012) previous finding that DT microbiomes are differentiated into four types was certainly supportive to our arguments for the niche selection hypothesis. Theoretically, Chisholm and Pacala (2010) provided an analytic proof that a strong niche model can generate exactly the same asymptotic form of SAD as the neutral model predicts. They suggested that, in this case, neutral theory should not be used to infer an absence of niche structure or to explain ecosystem functions (Chisholm and Pacala, 2010). Our case study here with the human DT microbiome provides an empirical mirror to Chisholm and Pacala (2010) theoretical study. In both cases, more realistic niche model is preferred to more idealized niche model.

It should be pointed out that, although we sided against the MSN inference ecologically at the metacommunity level, statistically we do not reject the validity of its model fitting including its parameter values. In particular, its algorithm for estimating the fundamental biodiversity numbers (θ) and fundamental dispersal (migration) numbers (*M*) with truly multisite SAD datasets is advantageous (Harris et al., 2017). For this reason, we even adopted the MSN model parameters in detecting a fifth niche previously.

More recently, Hammal et al. (2015) developed a power calculation method to quantify a model’s capacity to discern deviations from neutrality using SAD data. Their approach could have been a promising approach for us to resolving the previous overlap (conflict) between the MSN and NNH models. Nevertheless, to apply their approach, one has to extend their method to establish the NNH as an alternative hypothesis, which is beyond the scope of this study but is certainly worthy of the future studies. In addition, expanding alternative hypotheses to other highly relevant factors such as area (number of individuals in the case of human microbiomes), inter-individual heterogeneity, isolation, etc. may generate more powerful approaches for harnessing the insights from SAD data of the human microbiome. Therefore, we still believe SAD data provide rich opportunities to gain important knowledge on the mechanism underlying the diversity patterns of human microbiomes. Kadmon and Allouche (2007) offered an example for such efforts, which attempted to integrate two of the most important theories in community ecology—a unification of island biogeography and niche theory.

A second issue we would like to briefly discuss here is the assumed *stochastic* nature of dispersal and speciation. Among the four processes of Vellend–Hanson synthesis, while generally the drift was considered as stochastic and selection as deterministic, the nature of dispersal and speciation has been ambiguous. In the neutral theory of biodiversity, dispersal is assumed to be stochastic and equivalent among species. There are arguments to suggest that the neutral dispersal assumption should be rejected on principle because dispersal can be a species-specific process (property) and it evolves by natural selection (Clark, 2009; Lowe and McPeek, 2014). A discussion on the concept of speciation, which was considered as “mystery of mysteries” by Darwin, is beyond the scope of this article. In population genetics, selection is missing in some speciation models, which assumes a key role for stochastic events. Indeed, such models possess a long history in speciation research, but convincing evidence has been limited in practice (Safran and Nosil, 2012). The speciation concept in the neutral theory for biodiversity is treated as stochastic, but in reality, deterministic selection is very likely in effect too. Note that speciation in the neutral theory is measured in fundamental biodiversity (speciation) numbers, which is the *rate* at which new individuals are added to the metacommunity as a result of speciation, rather than as a result of reproduction. The concept is treated as stochastic in the formation of the neutral model. Obviously, this is an idealized treatment and whether or not the speciation is stochastic is likely similar to the debates in population genetics. For the previously discussed issues, the findings from this study can reveal less information on the role of speciation in Vellend–Hanson synthesis of four processes, other than being lumped together as a part of “stochastic drifts” (including drifts in demography, dispersal, and speciation). Therefore, our study could only offer some educated guess for the relative importance of the deterministic selection vs. stochastic drifts in shaping the microbiome diversity pattern along the DT, besides moderate insights on the existence of a fifth niche and the estimation of the microbial dispersal (migration) rate from oral to gut along the DT. Truly comprehensive, quantitative characterization of the niche-neutral continuum or the four-process paradigm is beyond the reach of this article. Our study may only be considered as a tiny step toward starting this important endeavor.

## Data Availability Statement

The original contributions presented in this study are included in the article/Supplementary Material, further inquiries can be directed to the corresponding author.

## Author Contributions

ZM designed and analyzed the data and wrote the manuscript. HC analyzed the data. Both authors contributed to the article and approved the submitted version.

## Conflict of Interest

The authors declare that the research was conducted in the absence of any commercial or financial relationships that could be construed as a potential conflict of interest.

## Publisher’s Note

All claims expressed in this article are solely those of the authors and do not necessarily represent those of their affiliated organizations, or those of the publisher, the editors and the reviewers. Any product that may be evaluated in this article, or claim that may be made by its manufacturer, is not guaranteed or endorsed by the publisher.
