# Supplementary material for: Niche-Neutral Continuum Seems to Explain the Global Niche Differentiation and Local Drift of the Human Digestive Tract Microbiome
Source: Front Microbiol. 2022 Jul 22;13:912240. doi: 10.3389/fmicb.2022.912240 (PMC9400020; doi:10.3389/fmicb.2022.912240)
Supplement: Supplementary file 1 [file Data_Sheet_1.PDF]

Online Supplementary Information (OSI) for: Chen & Ma (2021) Niche-neutral continuum explains the global niche differentiation and local drift of the human digestive tract microbiome.

The OSI includes Supplement to Materials and Methods as well as 10 Tables, with Tables S1 & S2 supplied in a separate MS-Excel file.

Supplement to "**Materials and Methods**" Section

Tang & Zhou (2013) niche-neutral hybrid model

Tang & Zhou (2013) proposed a hybrid niche-neutral model by revising Volkov et al. (2007) neutral model for multiple discrete communities. Volkov *et al.* (2007) assumed the inter-species interactions in a steady-state community can be ignored and all species in the community become functionally equivalent. They further assumed that birth and death probabilities of a species with n individuals are $b_n = b(n+\gamma)$ and $d_n = dn$ respectively, where b and d are the per-capita density-independent birth and death rates and γ is a parameter for immigration. The migration was assumed to be species-independent, corresponding to immigration from a time-averaged metacommunity in a species-symmetric manner. This treatment of migration, in effect, ignored any immigration between local communities within the metacommunity, and also the rates of immigration considered were small. By solving the master equation for the dynamics of a species, Volkov *et al.* (2007) obtained the probability that a species has n individuals, which follows the negative binomial distribution:

$$p(n) = \frac{(1-x)^{\gamma}}{\Gamma(\gamma)} \frac{x^n}{n!} \Gamma(n+\gamma) \qquad (1)$$

where x is the ratio of the *per* capita birth to death rate (*i.e.*, *b*/*d*, a measure of the lifetime reproductive success), and $\Gamma(z) = \int_0^{\infty} t^{z-1} e^{-t} dt$, which is equal to ($z$—1)! for integer z. They further obtained the *mean number of species* with abundance n:

$$< \varphi_n >= \theta \, \frac{x^n}{n!} \Gamma(n+\gamma) \qquad (2)$$

where θ is the *fundamental biodiversity parameter*.

Tang & Zhou assumed that a semi-isolated local community consists of K non-overlapping niches. Within each niche, a number of species follow their own neutral rules independent of the other K-1 niches. By applying Volkov *et al.* (2007) neutral model for multiple discrete communities to a single niche of the community, Tang & Zhou (2013) derived the expected number of species with abundance n in niche i as:

$$< \varphi_{n,i} >= \theta_i \frac{x_i^n}{n!} \Gamma(n+\gamma_i) \qquad (3)$$

where θ_i is the biodiversity parameter for niche i, x_i is the ratio of per capita birth to death rates of each species in niche i, and γ_i is a parameters for immigration of niche i. The total expected number of species with abundance n in the community consisting of K niches is represented by the following equation:

$$< \varphi_n ; K >= \sum_{i=1}^{K} < \varphi_{n,i} > \qquad (4)$$

Note that eqn. (4) is a summation of eqn. (3) across K niches, *i.e.*, summing up all species with abundance of n across all K niches. The following Chi-squared test statistic is utilized to determine the goodness-of-fitting for the niche-neutral hybrid model, *i.e.*,

$$\chi^2 = \sum_n \frac{(E_n - O_n)^2}{E_n} \tag{5}$$

where E_n is the expected number of species with abundance n, O_n is the observed number of species with abundance n.

In summary, a unique feature of Tang & Zhou (2013) niche-neutral hybrid model is its incorporation of niche differentiations into Volkov *et al*. (2007) multi-site neutral model. Specifically, the per capita birth to death rates (x) and immigration parameter (γ) vary among species from different niches. In the case of our multi-site microbiome datasets, we treat each site as a niche occupied by a local microbial community and fit the neutral model for each local community. By first computing the number of species with abundance n in each local community based on eqn. (3), and then computing total expected number of species with abundance n in the metacommunity based on eqn. (4), we can obtain the theoretically expected number of species with each abundance level ($n=1, 2,\ldots$). Finally, the Chi-squared test can be performed by comparing with the corresponding observed number of species for each corresponding abundance level (n) [eqn. (5)]. The p-value of Chi-squared test is then utilized to determine whether or not Tang & Zhou's (2013) hybrid model is suitable for a set of microbial communities sampled from the multiple microbiome sites of a human individual. Specifically, at the metacommunity level, if p-value>0.05, then the meta-community satisfies the NNH and the metacommunity assembly is co-driven by both niche and neutral processes, which also implies that the meta-community itself does not satisfy the neutral theory, but within each niche, the local community is neutral. If p-value<0.05, the metacommunity does not satisfy the NNH, which also implies that within each niche, the local community is not neutral either, and the metacommunity assembly is solely influenced by the niche process.

Harris *et al.* (2017) multisite neutral model (MSN)

We divide the description of Harris *et al*. (2017) MSN as the following four sections:

(*i*) *Hubbell's Unified Neutral Theory of Biodiversity and Biogeography (UNTB)*

The UNTB conceptually distinguishes local community dynamics from metacommunity dynamics, both of which are assumed to be driven by similar neutral processes (Hubbell 2001). Regarding the local community dynamics, assume there are M local communities indexed as i=1, 2... M, each with N_i individuals (N_i is considered constant for each local community). At each time step, the local community dynamics for site i is driven by a random process—selecting an individual randomly and either replacing it by a randomly chosen individual immigrated from the metacommunity with migration probability (m_i) or replacing it by a indigenous member randomly chosen from the local community (i) with probability (1-m_i). The UNTB further assumes that the local communities are at steady state, and each site is assigned a vector $\bar{\pi}_i=(\pi_{i,1},\ldots,\pi_{i,S})$, denoting the probability for observing a particular species at site i. The two parameters m_i and N_i can be replaced by a single parameter termed immigration rate, controlling the coupling of a local community to the metacommunity, *i.e.*,

$$I_i = (N_i - 1)[m_i/(1-m_i)]\cdot \tag{6}$$

Regarding the equivalent metacommunity neutral dynamics, new species are generated through speciation with a probability v. Similar to local community neutral dynamics, the speciation rate, also known as fundamental biodiversity number (θ), can be defined as:

$$\theta = (v/(1-v))(N-1), \tag{7}$$

where N is the fixed number of individuals in the metacommunity. The parameter θ can be interpreted as the *rate* at which new individuals are added to the metacommunity as a result of speciation.

The third aspect of the UNTB is to treat the observed community samples, *i.e*., the rows in the data matrix $\mathbf{X}_{MxS}$ with elements x_{ij} giving the abundance of species j is observed at site i, as a sample from the local community. As a side note, the matrix **X** is actually the OTU table of 16s-rRNA gene abundances in the case of test datasets we used in this study. Assume that the sample is taken with replacement, let $J_i = \sum_{j=1}^{S} x_{ij}$, and then the multinomial (MN) distribution describes the vector of observations at a given site, *i.e.,*

$$\overline{X}_i \sim MN(J_i, \overline{\pi}_i). \qquad (8)$$

(*ii*) *HDP (Hierarchical Dirichlet Process) Approximation to Neutral Metacommunities*
A fully general case of fitting multiple sites UNTB with different immigration rates is computationally rather challenging (actually intractable) even for small number of sites, and approximate algorithms must be utilized (Harris *et al*. 2017). Harris *et al*. (2017) developed an efficient Bayesian fitting framework by approximating the neutral models with the hierarchical Dirichlet process (HDP). Harris *et al*. (2017) approximation captures the essential elements of the UNTB, *i.e.,* neutrality, finite populations, and multiple panmictic geographically isolated populations linked by relatively rare migration—while little influenced by the specific details of the local community dynamics, as explained previously in (*i*).

Sloan *et al*. (2006, 2007) showed that for large local population sizes, assuming a fixed finite-dimensional metacommunity distribution with S species present, then the local community distribution, $\overline{\pi}_i$, can be approximated by a *Dirichlet* distribution (Sloan *et al* 2006 & 2007). But it was Harris *et al*. (2017) developed the general framework for approximating the UNTB computationally efficiently. Assuming there is a potentially infinite number of species that can be observed in the local community, then the steady state distribution of observing local population i is a Dirichlet process (DP), *i.e*.,

$$\overline{\pi}_i \mid I_i, \overline{\beta} \sim DP(I_i, \overline{\beta}) \qquad (9)$$

where $\overline{\beta} = (\beta_1, ..., \beta_S)$ is the relative frequency of each species in the metacommunity.

At the metacommunity level, a Dirichlet process is still applicable, but then the base distribution is simply a uniform distribution over arbitrary species labels, and the concentration parameter is the biodiversity parameter (θ) (Harris *et al*. 2017). This observation was also implicit in early fitting of the neutral models (Etienne 2005). The metacommunity distribution is purely the stick breaking process, *i.e*.,

$$\overline{\beta} \sim Stick(\theta). \qquad (10)$$

Given that both local community and metacommunity are Dirichlet processes, it becomes a hierarchical Dirichlet process (HDP) in the domain of machine learning (Teh *et al.* 2006, Harris *et al*. 2017).

Alternatively, Dirichlet process (DP) can also be viewed as the so-termed Chinese restaurant process, from which the Antoniak equation can be derived. Antoniak equation represents the number of species (S) observed following N draws from a DP with concentration parameter θ and is in the following form:

$$P(S \mid \theta, N) = s(N, S)\theta^S \frac{\Gamma(\theta)}{\Gamma(\theta + N)} \qquad (11)$$

where $s(N, S)$ is the unsigned Stirling number of the first kind and $\Gamma(.)$ denotes the gamma function (Antoniak 1974, Harris *et al* 2017).

(*iii*) *Gibbs Sampler (MCMC algorithm) for the HDP-MSN model*
The full neutral-HDP model is formed by combining previous equations (8-10). Although the

potential number of species is infinite because the evolutionary process may generate new species continuously, the observed number of species during a period is certainly finite. The following derivation and interpretation were based on Harris *et al.* (2017).

Assume that the proportions of the observed species (S) are β_k (k=1,...,S) and the unrepresented component is $\beta_u = \sum_{k=S+1}^{L} \beta_k (L \to \infty)$, then the species abundance distributions (SADs) in the local communities can be represented as:

$$\overline{\pi}_i \sim DP(I_i\beta_1,...,I_i\beta_S,I_i\beta_u) \tag{12}$$

From eqn. (12), one can then marginalize the local community distributions and obtain the probability of the observed frequencies given the metacommunity distribution $\overline{\beta}$ and the immigration rates I_i, for each local community (i=1,..., M):

$$P(X \mid \overline{\beta},I_1,...,I_M) = \prod_{i=1}^{M} \frac{J_i!}{X_{i1}!...X_{iS}!} \frac{\Gamma(I_i)}{\Gamma(J_i+I_i)} \prod_{j=1}^{S} \frac{\Gamma(x_{ij}+I_i\beta_i)}{\Gamma(I_i\beta_i)}. \tag{13}$$

Harris et al. (2017) devised an efficient Gibbs sampler for the UNTB-HDP approximation, which is a type of Bayesian Markov Chain Monte Carlo (MCMC) algorithm and can be summarized as the following four sampling steps:

(***a***) Sample the biodiversity paramete*r* (θ) from the conditional

$$P(\theta \mid S,T) \propto s(T,S)\theta^S \frac{\Gamma(\theta)}{\Gamma(\theta+T)} Gamma(\theta \mid \alpha,\zeta) \tag{14}$$

where θ is the biodiversity parameter. $T = \sum_{i=1}^{M}\sum_{j=1}^{S} T_{ij}$ is the number of ancestors, S is the number of species in metacommunity, $s(T, S)$ is the unsigned Stirling number of the first kind(Antoniak 1974), α and ζ are constants.

(***b***) Sample the metacommunity distribution

$$\overline{\beta} = (\beta_1,\beta_2...,\beta_S,\beta_u) \sim DP(T_{\cdot 1},T_{\cdot 2},...,T_{\cdot S},\theta) \tag{15}$$

where $T_{\cdot j} = \sum_{i=1}^{M} T_{ij}$ is the number of ancestors of species j in metacommunity.

(***c***) Sample the immigration rates

$$P(I_i \mid T_{ij}) \propto \frac{\Gamma(I_i)}{\Gamma(J_i+I_i)} I_i^{T_i} Gamma(I_i \mid \eta,\nu) \tag{16}$$

where both η and ν are constants.

(***d***) Sample the ancestral states

$$P(T_{ij} \mid x_{ij},I_i,\beta_i) = \frac{\Gamma(I_i\beta_j)}{\Gamma(x_{ij}+I_i\beta_j)} s(x_{ij},T_{ij})(I_i\beta_j)^{T_{ij}} \tag{17}$$

where the various symbols have the same representations as previously defined.

Harris *et al*. (2017) found through experiments that to ensure sampling was from the steady state distribution, 50,000 Gibb samples for each fitted dataset were required with the first 25,000 iterations removed as burn-in. The results are reported as the median values over the last 25,000

samples with upper and lower credible limits (Bayesian confidence) given by 2.5% and 97.5% quantiles of those samples.

(*iv*) *Goodness-of-fitting test for the HDP-MSN model*
To judge whether an observed dataset fits to the HDP-neutral model, Harris *et al.* (2017) proposed a similar Monte Carlo significance test to that used by Etienne (2007). Assuming that the *k-th* posterior sample of fitted UNTB parameters are $\theta^k, I_1^k, ... I_M^k$ for this dataset, an artificial (simulated) data matrix with the same number of samples M and the same sample sizes J_i as the original data matrix is simulated and generated by sampling from the full neutral-HDP, denote by $\mathbf{X}_0^k$.

In the meantime, for the same dataset, one can also generate a neutral metacommunity distribution, $\overline{\beta}_0^k$, using equation (17), since the ancestral frequencies $T_{\cdot j} = \sum_{i=1}^{M} T_{ij}$ are known. We then calculate the likelihood $P(X_0^k \mid \overline{\beta}_0^k, I_1^k, ..., I_M^k)$ using equation (13). These likelihoods were then compared with the actual likelihood of the observed sample, *i.e.*, $P(X \mid \overline{\beta}^k, I_1^k, ..., I_M^k)$. A pseudo p-value, denoted by p_M, can be computed from the comparisons, and is then harnessed to determine if the dataset satisfied with the neutral model.

Furthermore, Harris *et al* (2017) also developed a procedure to test for local neutral community assembly but with a fitted potentially non-neutral metacommunity because of the hierarchical nature of the model. The procedure started with the generation of datasets, $\boldsymbol{X}_1^k$, with the metacommunity fixed at the model fitted values, $\overline{\beta}^k$. The likelihood for each of the samples, $P(X_1^k \mid \overline{\beta}^k, I_1^k, ..., I_M^k)$, is computed and compared to $P(X \mid \overline{\beta}^k, I_1^k, ..., I_M^k)$. The proportion of samples having likelihood greater than this forms the pseudo p-value, denoted by p_L is then utilized for testing the neutrality of the local community assembly. For both local and metacommunity level tests, samples were generated from 2500 sets of fitted parameters sampled from every tenth iteration of the last 25000 Gibbs samples (the first 25000 samples were removed as the burn-in, and a total of 50000 Gibb samples were simulated).

References

Etienne RS (2005) A new sampling formula for neutral biodiversity. *Ecology Letters*, 8(3):253-260.

Etienne RS (2007) A neutral sampling formula for multiple samples and an 'exact' test of neutrality. *Ecology Letters*, 10(7):608-618.

Harris K, Parsons TL, Ijaz UZ, et al. (2017). Linking Statistical and Ecological Theory: Hubbell's Unified Neutral Theory of Biodiversity as a Hierarchical Dirichlet Process. *Proceedings of the IEEE*, 105(3):516-529.

Hubbell, Stephen P (2001) The unified neutral theory of biodiversity and biogeography. *Princeton University Press*

Sloan W, Lunn M, Woodcock S, et al. (2006). Quantifying the roles of immigration and chance in shaping prokaryote community structure. *Environmental Microbiology*, 8(4):732-740.

Sloan WT, Woodcock S, Lunn M, et al. (2007). Modeling taxa-abundance distributions in

microbial communities using environmental sequence data. *Microbial Ecology*, 53(3):443-455.

Tang J, Zhou S. (2013). Hybrid niche-neutral models outperform an otherwise equivalent neutral model for fitting coral reef data. *Journal of Theoretical Biology*, 317(1):212-218.

Teh YW, Jordan MI, Beal MJ, et al. (2006). Hierarchical Dirichlet Processes. *Journal of the American Statistical Association*, vol. 101(476):1566-1581.

Volkov I, Banavar JR, Hubbell SP, Maritan A. (2007). Patterns of relative species abundance in rainforests and coral reefs. *Nature* **450**:45–49.

Supplementary Tables (S1-S10)

Table S1. The results of fitting the HDP-MSN (hierarchical Dirichlet process, multi-site neutral) model with 1000 times of re-sampling to the human DT datasets (*MS-Excel Table*)

Legends to Tables S1, S6 and S8 are the same as those for Table 1 in the main text, except for two additional columns. Due to type error in Harris et al. (2017), the $\boldsymbol{P_M}$-values should be adjusted as ($\boldsymbol{P_M}$=1−$\boldsymbol{P_{MS}}$), where $\boldsymbol{P_{MS}}$ is output from their computational program. Similarly, the $\boldsymbol{P_L}$-values should be adjusted as ($\boldsymbol{P_L}$=1−$\boldsymbol{P_{LS}}$), where $\boldsymbol{P_{LS}}$ is output from their computational program. In other words, 1's complement of computed *P*-values should be used for the testing.

Table S2. The results of fitting Tang & Zhou's (2013) NNH (niche-neutral hybrid) model with 1000 times of re-sampling to the human DT datasets (*MS-Excel Table*)

Table S3. The passing percentages from testing the MSN (multi-site neutral model) and NNH (niche-neutral hybrid model) with the human DT microbiome datasets, summarized from Tables S1-S2

***Samples* (*N*)**	**MSN (Multi-Site Neutral)**				**NNH (Niche-Neutral Hybrid)**			
	**Metacommunity**		**Local Community**		**Metacommunity**		**Local Community**	
	***N* (pass)**	**%**	***N* (pass)**	**%**	***N***	**%**	**Avg. *N* (Pass)**	**Avg. %**
1000	1000	100	1000	100	921	92.1	5.6	95.9

Table S4. The p-value from fitting the power law and normal distribution to the key parameters of the MSN and NNH models (p>0.05 indicating significant fitting)*

**Statistical Distribution**	**MSN (multisite neutral) model**			**NNH (niche-neutral hybrid) model**			
	θ	***m***	***M***	***θ***	***m***	***x***	**γ**
Normal distribution (p-value)	<0.001	<0.001	<0.001	<0.001	<0.001	0.014	<0.003
Power law distribution (p-value)	0.364	0.999	0.343	0.117	0.122	0.219	0.667
Power law distribution parameter (*K*)	14.186	5.499	8.434	7.199	5.901	38.511	11.793

*The power law distribution is described with: $p(X = x) = \frac{K-1}{x_{\min}}\left(\frac{x}{x_{\min}}\right)^{-K} \quad (K > 0)$

*The R-function: shapiro.test and power.law.fit in R packages were used to fit the distribution. All key MSN/NNH parameters successfully fitted to the power law distribution but failed to fit to the Normal distribution.

Table S5. Comparative summary of the performances of MSN and NNH models fitted to the human DT microbiome datasets of 1000 random samples (meta-communities), summarized from Tables S1-S2

**Samples (*N*)**	**MSN only**		**NNH only**		**Both MSN & NNH**		**NOT** (MSN, NNH)	
	N	**%**	***N***	**%**	***N***	**%**	***N***	**%**
1000	79	7.9%	0	0	921	92.1	0	0

Table S6. Fitting the HDP-MSN (hierarchical *Dirichlet* process, multi-site neutral) model to the pair-wised (pair of sites) microbiome samples (from the 10 DT sites) with 100 times of re-sampling*

Pair of Sites	L_O	θ	***M-value***	**Meta-community (Body)**					**Local Community (DT Site)**				
				L_M	N_{MS}	N	P_{MS}	P_M=1-P_{MS}	L_L	N_{LS}	N	P_{LS}	P_L=1-P_{LS}
BM-HP	-526.892	22.217	54.729	-612.226	408.05	2500	0.163	0.837	-546.962	834.97	2500	0.334	0.666
BM-KG	-426.254	20.421	34.258	-496.233	456.94	2500	0.183	0.817	-447.978	809.79	2500	0.324	0.676
BM-PT	-533.601	25.404	37.067	-588.659	658.14	2500	0.263	0.737	-555.297	810.35	2500	0.324	0.676
BM-Sal	-556.876	27.020	37.526	-591.584	854.78	2500	0.342	0.658	-574.290	898.45	2500	0.359	0.641
BM-Stool	-627.399	160.408	6.812	-601.013	1582.82	2500	0.633	0.367	-620.470	1339.97	2500	0.536	0.464
BM-SubP	-569.280	29.947	22.576	-593.537	970.05	2500	0.388	0.612	-590.032	849.84	2500	0.340	0.660
BM-SupP	-541.482	27.570	24.390	-572.612	877.74	2500	0.351	0.649	-563.375	825.77	2500	0.330	0.670
BM-TD	-505.420	23.854	32.435	-552.585	698.32	2500	0.279	0.721	-526.002	825.67	2500	0.330	0.670
BM-Th	-547.820	26.446	42.293	-608.750	642.94	2500	0.257	0.743	-567.767	863.39	2500	0.345	0.655
HP-KG	-452.828	23.605	34.522	-511.873	568.52	2500	0.227	0.773	-471.531	882.01	2500	0.353	0.647
HP-PT	-548.050	24.579	45.674	-599.835	683.87	2500	0.274	0.726	-565.181	901.23	2500	0.360	0.640
HP-Sal	-581.686	25.762	52.799	-628.471	733.96	2500	0.294	0.706	-596.501	952.89	2500	0.381	0.619
HP-Stool	-660.469	154.490	7.638	-628.121	1645.9	2500	0.658	0.342	-649.130	1394.92	2500	0.558	0.442
HP-SubP	-604.947	33.042	24.019	-629.051	972.12	2500	0.389	0.611	-623.210	918.9	2500	0.368	0.632
HP-SupP	-569.860	30.121	24.469	-598.771	897.12	2500	0.359	0.641	-587.183	922.17	2500	0.369	0.631
HP-TD	-519.537	21.778	46.248	-572.704	677.38	2500	0.271	0.729	-536.526	893.44	2500	0.357	0.643
HP-Th	-564.696	25.732	51.737	-622.413	648.68	2500	0.259	0.741	-581.016	922.17	2500	0.369	0.631
KG-PT	-453.694	24.768	26.215	-496.560	744.74	2500	0.298	0.702	-472.451	879.46	2500	0.352	0.648
KG-Sal	-477.154	27.024	33.259	-503.418	907.75	2500	0.363	0.637	-489.778	1003.4	2500	0.401	0.599
KG-Stool	-521.251	144.353	5.254	-497.143	1579.8	2500	0.632	0.368	-512.883	1374.66	2500	0.550	0.450
KG-SubP	-480.002	29.549	17.585	-489.810	1128.32	2500	0.451	0.549	-492.770	1003.26	2500	0.401	0.599
KG-SupP	-465.437	27.671	15.737	-478.380	1073.56	2500	0.429	0.571	-480.999	939.77	2500	0.376	0.624
KG-TD	-423.895	23.001	20.380	-449.852	907.99	2500	0.363	0.637	-441.558	889.16	2500	0.356	0.644
KG-Th	-470.625	27.304	27.641	-512.430	748.95	2500	0.300	0.700	-488.016	921.42	2500	0.369	0.631
PT-Sal	-579.823	23.527	56.809	-622.791	769.53	2500	0.308	0.692	-594.286	956.03	2500	0.382	0.618
PT-Stool	-688.898	162.747	7.334	-649.898	1710.21	2500	0.684	0.316	-671.103	1473.15	2500	0.589	0.411
PT-SubP	-611.030	29.826	28.065	-639.118	929.45	2500	0.372	0.628	-628.345	937.4	2500	0.375	0.625
PT-SupP	-587.927	30.560	22.497	-608.838	999.22	2500	0.400	0.600	-605.802	914.9	2500	0.366	0.634
PT-TD	-526.816	21.181	46.811	-569.332	774.5	2500	0.310	0.690	-543.159	917.04	2500	0.367	0.633
PT-Th	-566.281	24.437	48.501	-617.547	713.33	2500	0.285	0.715	-582.801	917.58	2500	0.367	0.633
Sal-Stool	-700.979	152.834	8.225	-654.848	1802.5	2500	0.721	0.279	-677.516	1557.51	2500	0.623	0.377
Sal-SubP	-602.590	29.718	33.385	-622.760	1028.67	2500	0.411	0.589	-613.716	1032.48	2500	0.413	0.587
Sal-SupP	-576.875	30.506	29.056	-590.582	1086.71	2500	0.435	0.565	-587.401	1043.91	2500	0.418	0.582
Sal-TD	-537.910	23.074	56.638	-576.345	816.85	2500	0.327	0.673	-548.342	1024.06	2500	0.410	0.590
Sal-Th	-592.407	28.773	48.526	-634.175	796.27	2500	0.319	0.681	-606.957	968.47	2500	0.387	0.613
SubP-Stool	-703.334	170.057	7.386	-653.999	1819.06	2500	0.728	0.272	-675.364	1598.81	2500	0.640	0.360
SubP-SupP	-540.851	21.484	48.625	-563.597	991.46	2500	0.397	0.603	-551.414	1027.43	2500	0.411	0.589
SubP-TD	-557.766	27.515	22.553	-576.311	1027.44	2500	0.411	0.589	-572.132	967.83	2500	0.387	0.613
SubP-Th	-598.781	31.145	25.720	-623.522	978.54	2500	0.391	0.609	-613.903	962.46	2500	0.385	0.615
SupP-Stool	-673.851	176.053	6.819	-624.563	1823.69	2500	0.729	0.271	-644.753	1611.14	2500	0.644	0.356
SupP-TD	-530.841	25.985	20.433	-547.438	1053.51	2500	0.421	0.579	-544.341	981.71	2500	0.393	0.607
SupP-Th	-579.619	32.081	22.486	-599.408	1008.33	2500	0.403	0.597	-594.219	973.68	2500	0.389	0.611
TD-Stool	-634.710	155.866	6.658	-592.934	1762.05	2500	0.705	0.295	-611.791	1552.54	2500	0.621	0.379
TD-Th	-536.884	23.322	50.616	-583.663	763.6	2500	0.305	0.695	-551.222	956.08	2500	0.382	0.618
Th-Stool	-673.175	145.477	7.833	-636.416	1701.05	2500	0.680	0.320	-657.968	1455.8	2500	0.582	0.418
**Mean**	-560.678	52.716	29.561	-582.758	1009	2500	0.404	0.596	-570.165	1037	2500	0.415	0.585
**Std. Err.**	10.441	7.979	2.381	7.838	58.49	0	0.023	0.023	8.989	35.32	0	0.014	0.014

The table legends here are the same as those for Table 1. P_M and P_L should be used for testing the MSN model. P_{MS} and P_{LS} are computed from Harris *et al*. (2017) program and need to be corrected with *1*'s complement.

Table S7. Fitting the NNH (niche-neutral hybrid) model to the pair-wised (pair of sites) microbiome samples (from the 10 DT sites) with 100 times of re-sampling; unlike in Table S1 & S2, here the parameters for each pair of sites are the averages from 100 times of re-sampling.

Pair of Sites	J	S	θ	m	x	γ	R^2	χ^2	p-value	N^{pass}	$\%^{(pass)}$
BM-HP	11237.310	50.914	12.005	0.0002	0.815	1.130	0.776	8.147	0.672	1.598	79.9
BM-KG	11396.017	41.652	11.044	0.0002	0.795	1.168	0.750	76.852	0.649	1.348	67.4
BM-PT	10754.780	48.758	11.210	0.0002	0.806	1.236	0.735	7.613	0.702	1.429	71.4
BM-Sal	10540.629	51.775	10.295	0.0003	0.811	1.309	0.703	7.816	0.676	1.461	73.0
BM-SubP	11262.631	49.060	10.410	0.0002	0.807	1.281	0.705	7.834	0.710	1.286	64.3
BM-SupP	11448.098	48.030	10.392	0.0002	0.803	1.282	0.712	10.284	0.671	1.378	68.9
BM-TD	11033.217	46.161	10.289	0.0002	0.810	1.254	0.705	7.678	0.701	1.411	70.6
BM-Th	12190.039	51.837	11.588	0.0002	0.816	1.193	0.738	8.231	0.657	1.517	75.8
BM-Stool	13009.821	44.500	10.769	0.0002	0.798	1.211	0.754	10.008	0.644	1.457	72.8
HP-KG	10811.571	45.371	10.889	0.0002	0.804	1.186	0.687	63.489	0.560	1.247	62.4
HP-PT	10789.861	51.711	10.128	0.0002	0.803	1.357	0.700	8.521	0.623	1.478	73.9
HP-Sal	10003.265	55.671	10.755	0.0002	0.817	1.277	0.720	8.471	0.624	1.447	72.4
HP-SubP	11730.595	53.821	10.109	0.0002	0.805	1.364	0.678	9.290	0.608	1.298	64.9
HP-SupP	10136.447	50.165	9.420	0.0003	0.801	1.383	0.658	8.242	0.641	1.282	64.1
HP-TD	10551.229	49.441	10.157	0.0002	0.820	1.255	0.667	8.849	0.595	1.400	70.0
HP-Th	9817.688	54.392	11.762	0.0003	0.804	1.249	0.740	8.705	0.629	1.466	73.3
HP-Stool	11948.810	48.429	11.031	0.0002	0.807	1.198	0.724	10.342	0.558	1.359	67.9
KG-PT	11660.747	41.012	9.457	0.0003	0.777	1.382	0.651	55.985	0.622	1.259	63.0
KG-Sal	10650.154	45.068	9.279	0.0003	0.787	1.385	0.677	58.800	0.585	1.333	66.7
KG-SubP	11661.082	40.551	7.992	0.0003	0.769	1.513	0.614	57.433	0.635	1.228	61.4
KG-SupP	11702.563	38.569	8.136	0.0003	0.778	1.416	0.632	58.936	0.613	1.200	60.0
KG-TD	11954.525	36.734	8.502	0.0003	0.784	1.403	0.568	64.525	0.596	1.253	62.7
KG-Th	12341.924	45.116	10.054	0.0003	0.777	1.363	0.710	60.455	0.597	1.337	66.9
KG-Stool	12169.268	36.762	9.276	0.0003	0.782	1.284	0.709	41.312	0.636	1.333	66.7
PT-Sal	10024.835	53.229	9.573	0.0003	0.801	1.458	0.632	9.035	0.577	1.365	68.2
PT-SubP	11408.562	51.475	8.947	0.0003	0.792	1.540	0.622	8.652	0.654	1.309	65.4
PT-SupP	11383.829	50.203	8.710	0.0002	0.786	1.536	0.597	8.598	0.617	1.241	62.0
PT-TD	11163.298	48.298	8.782	0.0003	0.795	1.506	0.591	8.970	0.603	1.369	68.5
PT-Th	10511.818	53.159	10.493	0.0002	0.805	1.348	0.691	8.613	0.629	1.412	70.6
PT-Stool	12447.954	46.937	9.599	0.0002	0.789	1.395	0.696	9.621	0.607	1.483	74.1
Sal-SubP	8933.006	53.648	7.606	0.0004	0.778	1.683	0.590	8.000	0.629	1.272	63.6
Sal-SupP	7828.900	51.800	7.477	0.0004	0.780	1.631	0.600	10.557	0.624	1.313	65.6
Sal-TD	8932.205	51.337	8.028	0.0003	0.785	1.587	0.598	8.107	0.642	1.301	65.1
Sal-Th	9467.083	55.280	10.117	0.0003	0.796	1.431	0.692	8.015	0.646	1.405	70.2
Sal-Stool	10361.691	48.163	8.463	0.0003	0.787	1.473	0.685	9.165	0.625	1.382	69.1
SubP-SupP	10036.415	47.732	6.788	0.0003	0.781	1.660	0.540	11.357	0.649	1.183	59.2
SubP-TD	10331.125	46.074	7.663	0.0003	0.783	1.580	0.608	9.108	0.601	1.353	67.6
SubP-Th	9681.306	51.706	9.660	0.0003	0.791	1.445	0.651	8.777	0.632	1.263	63.1
SubP-Stool	11305.959	43.471	8.474	0.0003	0.793	1.387	0.650	9.826	0.623	1.259	62.9
SupP-TD	9935.307	44.436	6.468	0.0003	0.788	1.632	0.547	8.093	0.651	1.314	65.7
SupP-Th	9065.460	50.358	8.916	0.0003	0.794	1.474	0.634	7.615	0.669	1.239	61.9
SupP-Stool	11663.644	43.561	8.249	0.0002	0.793	1.409	0.653	8.788	0.637	1.278	63.9
TD-Th	10929.637	50.863	9.240	0.0002	0.790	1.475	0.672	8.551	0.632	1.357	67.9
TD-Stool	10277.713	41.945	7.375	0.0003	0.790	1.498	0.665	8.279	0.659	1.439	72.0
Th-Stool	11548.600	47.628	10.275	0.0003	0.792	1.349	0.713	9.952	0.595	1.400	70.0
**Mean**	10845.347	47.929	9.463	0.0003	0.795	1.391	0.668	19.011	0.631	1.350	67.5
**Std. Err.**	158.745	0.719	0.201	0.0000	0.002	0.021	0.008	3.130	0.005	0.014	0.7

Table S8. The mean model parameters for the pair-wise MSN model between gut and 9 oral sites (*i.e.*, pair of gut and one of the 9 oral sites) excerpted from Table S6

Pair of Sites	L_O	θ	*M-value*	Meta-community (Body)					Local Community (DT Site)				
				L_M	N_{MS}	N	P_{MS}	P_M=1-P_{MS}	L_L	N_{LS}	N	P_{LS}	P_L=1-P_{LS}
BM-Stool	-627.399	160.408	6.812	-601.013	1582.82	2500	0.633	0.367	-620.470	1339.97	2500	0.536	0.464
HP-Stool	-660.469	154.490	7.638	-628.121	1645.9	2500	0.658	0.342	-649.130	1394.92	2500	0.558	0.442
KG-Stool	-521.251	144.353	5.254	-497.143	1579.8	2500	0.632	0.368	-512.883	1374.66	2500	0.550	0.450
PT-Stool	-688.898	162.747	7.334	-649.898	1710.21	2500	0.684	0.316	-671.103	1473.15	2500	0.589	0.411
Sal-Stool	-700.979	152.834	8.225	-654.848	1802.5	2500	0.721	0.279	-677.516	1557.51	2500	0.623	0.377
SubP-Stool	-703.334	170.057	7.386	-653.999	1819.06	2500	0.728	0.272	-675.364	1598.81	2500	0.640	0.360
SupP-Stool	-673.851	176.053	6.819	-624.563	1823.69	2500	0.729	0.271	-644.753	1611.14	2500	0.644	0.356
TD-Stool	-634.710	155.866	6.658	-592.934	1762.05	2500	0.705	0.295	-611.791	1552.54	2500	0.621	0.379
Th-Stool	-673.175	145.477	7.833	-636.416	1701.05	2500	0.680	0.320	-657.968	1455.8	2500	0.582	0.418

The table legends here are the same as those for Table 1. P_M and P_L should be used for testing the MSN model. P_{MS} and P_{LS} are computed from Harris *et al*. (2017) program and need to be corrected with *1*'s complement.

Table S9. The mean model parameters for the pair-wise NNH model between gut and 9 oral sites (*i.e.*, pair of gut and one of the 9 oral sites) excerpted from Table S7

Pair of Sites	*J*	*S*	θ	*m*	*x*	γ	R^2	χ^2	*p*-value	N^{pass}	$\%^{(pass)}$
BM-Stool	13009.821	44.500	10.769	0.0002	0.798	1.211	0.754	10.008	0.644	1.457	72.8
HP-Stool	11948.810	48.429	11.031	0.0002	0.807	1.198	0.724	10.342	0.558	1.359	67.9
KG-Stool	12169.268	36.762	9.276	0.0003	0.782	1.284	0.709	41.312	0.636	1.333	66.7
PT-Stool	12447.954	46.937	9.599	0.0002	0.789	1.395	0.696	9.621	0.607	1.483	74.1
Sal-Stool	10361.691	48.163	8.463	0.0003	0.787	1.473	0.685	9.165	0.625	1.382	69.1
SubP-Stool	11305.959	43.471	8.474	0.0003	0.793	1.387	0.650	9.826	0.623	1.259	62.9
SupP-Stool	11663.644	43.561	8.249	0.0002	0.793	1.409	0.653	8.788	0.637	1.278	63.9
TD-Stool	10277.713	41.945	7.375	0.0003	0.790	1.498	0.665	8.279	0.659	1.439	72.0
Th-Stool	11548.600	47.628	10.275	0.0003	0.792	1.349	0.713	9.952	0.595	1.400	70.0

Table S10. The mean model parameters for the pair-wise MSN/NNH models for *each* site: *i.e.*, each site was involved pair-wisely with 9 other sites, and also 9 MSN/NNH models. For each site, the average model parameters were computed from the 9 MSN/NNH models.

DT Site	MSN		NNH			
	θ	*M*	θ	*m*	*x*	γ
BM	36.329	29.209	9.800	0.0002	0.726	1.106
HP	36.133	34.184	9.626	0.0002	0.728	1.140
KG	34.770	21.485	8.463	0.0002	0.705	1.210
Sal	36.824	35.622	8.159	0.0003	0.714	1.323
PT	36.703	31.897	8.690	0.0002	0.715	1.276
Th	36.472	32.535	9.210	0.0002	0.716	1.233
TD	34.558	30.277	7.650	0.0003	0.714	1.319
SubP	40.228	22.991	7.765	0.0003	0.710	1.345
SupP	40.203	21.451	7.456	0.0003	0.710	1.342
Stool	142.229	6.396	8.351	0.0002	0.713	1.220
